# An evo-devo perspective on root genetic variation in cereals

**DOI:** 10.1093/jxb/erw505

**Published:** 2017-02-15

**Authors:** Silvio Salvi

**Affiliations:** 1 Department of Agricultural Sciences, University of Bologna, Italy

**Keywords:** Embryo transcriptome, maize (*Zea mays*), non-syntenic genes, QTLome, RNA-Seq, root primordia, *rtcs*, RTCS-dependent regulation, seminal root.

Evolutionary developmental biology (evo-devo) deals with the identification of genetic events underlying the evolution of morphological diversity – a complex task. A new paper in *Journal of Experimental Botany* by Tai *et al.* (pages 403–414) provides evidence that evolutionarily young non-syntenic genes were involved in the appearance of seminal roots in the maize evolutionary lineage.

From a strictly developmental perspective, and ignoring environmental effects, a morphological feature (such as an organ) is the result of developmental events that initially occur at the level of one or a few cells. These cells integrate external and endogenous signals, and then the first step on the developmental pathway is a change in gene expression. This is followed by division, growth, differentiation, and/or programmed cell death. Resolving the appearance of a morphological trait from an evolutionary developmental (evo-devo) perspective remains a more elusive proposition (Carroll, 2008; de Bruijn *et al.*, 2012). In this case, the genetic events (mutations) which occurred in the evolutionary lineages and led to large developmental changes have to be identified and arranged in temporal order. The same events should also be considered in terms of natural selection and genetic drift in order to explain their diffusion into the population. In any case, it is well recognized that the molecular genetics of the developmental processes under investigation should first be fully deciphered (Carroll, 2008).

The development of a specific root-type in maize – the seminal roots (Box 1) – is a useful model in an evo-devo context for a number of reasons. First, seminal roots evolved in the teosinte (the wild ancestor of cultivated maize)/maize lineage quite recently and after divergence from sorghum, which is closely related but lacks this type of root (Singh *et al.*, 2010). Second, high-quality genome sequences (Paterson *et al.*, 2009; Schnable *et al.*, 2009) and well-described synteny relationships (Wei *et al.*, 2007) are available for both species, which makes it possible to use comparative genomics to identify mutations of all types that occurred after the two species diverged.

**Box 1. Schematic representation of embryonic and early post-embryonic root systems of maize, rice, sorghum and wheat**

In the four species, a distinguishable primary root (PR) is the first root to emerge at the base of the embryo. Overall, rice and sorghum have a similar architecture with the first nodal roots (NR) or crown roots (also called ‘embryonic’ crown roots in rice; Rebouillat *et al.*, 2009) developing at the coleoptilar node (CN). Maize shares the same architecture but develops a variable number (0–13; Hochholdinger, 2009) of seminal roots (SR), which emerge from the scutellar node (SN) both dorsally and ventrally relative to the shoot (Salvi *et al.*, 2016). Four to five seminal roots also develop in wheat, next to its primary root. Rice, sorghum and maize share a variably elongated mesocotyl (M), which is anatomically a root–stem hybrid structure connecting the scutellum with the coleoptilar node. In these species, mesocotyl elongation pushes the young shoot tip to emerge from the soil. In wheat, instead, it is the first internode (also called the subcrown internode, SI) which usually elongates. For all species, a large number of nodal (crown) roots develop at the basal nodes of the stem later in development (not shown, with the exception of the first emerging nodal roots in wheat). All types of root discussed above develop lateral roots (LR). C, coleoptile.

**Figure:**
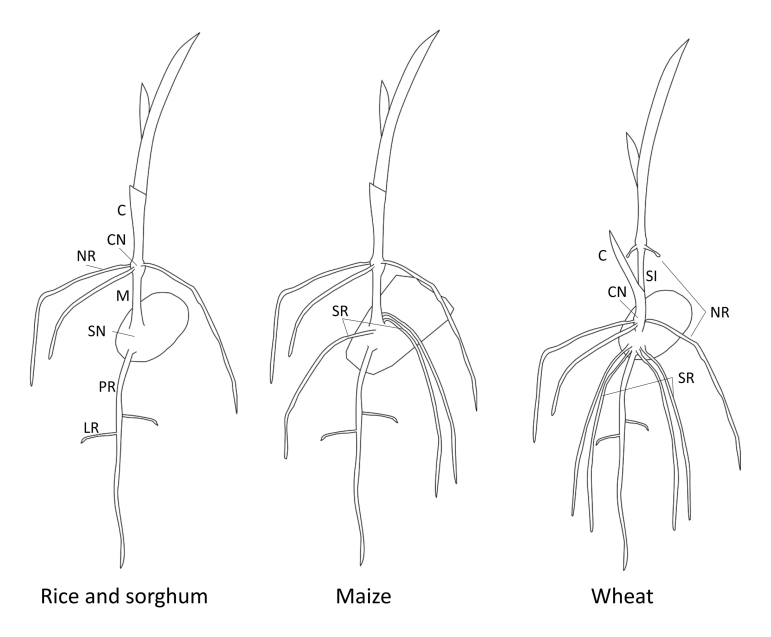

The teosinte/maize and sorghum lineages diverged about 12 million years ago from a ten-chromosome ancestor, and between 12 and 5 million years ago a tetraploidization – a whole-genome duplication either by auto or allopolyploidy (Schnable *et al.*, 2009) – occurred in the teosinte/maize lineage only. This event was followed by a massive genome rearrangement which eventually resulted, again, in a ten-chromosome genome where two subgenomes can still be distinguished (Wei *et al.*, 2007). At the same time, the genome also underwent a significant expansion caused by massive transposon activity (SanMiguel *et al.*, 1998). Altogether, this provides the perfect scenario for an evo-devo type of investigation.

## Developmental genetics of the maize root system

Maize has a complex root system including a primary root (or radicle) that develops directly from the basal end of the embryo, seminal roots which are initiated from the scutellar node, and nodal (or shoot-borne) roots (called crown or brace, if below or above the soil level, respectively; see Box 1 in Hochholdinger, 2016) developing from the lower nodes of the stem. All these roots also carry lateral roots. Depending on the genotype and growing conditions, primary and seminal roots may persist throughout the life cycle of the plant or die as the seedling develops shoot-borne roots (Hochholdinger and Tuberosa, 2009). The maize mutants *rootless concerning crown and seminal roots* (*rtcs*) and *rootless with undetectable meristems 1* (*rum1*) (both involved in auxin signalling) lack seminal root initiation but not in an organ-specific fashion: *rtcs* also lacks shoot-borne roots, whereas *rum1* does not show any lateral root development from the primary root in addition to the seminal root defect (Hochholdinger, 2009). Quantitative trait loci (QTLs) for seminal root number and architecture have been mapped in correspondence with these two loci and in other genome locations (Kumar *et al.*, 2012; Zhang *et al.*, 2013; Salvi *et al.*, 2016).

Variation for seminal root traits has adaptive potential. Different seminal root architectures appear to affect early crop establishment and P acquisition (Zhu *et al.*, 2005; Lynch, 2013; Mi *et al.*, 2016) (see also Gao and Lynch, 2016; Hochholdinger, 2016). Seminal roots also have unique anatomical features which are suggestive of higher absorption efficiency by comparison with other root types (Tai *et al.*, 2016). Thus, it is not surprising that there may have been positive selection for number of seminal roots during maize domestication and improvement (Burton *et al.*, 2013).

## Evo-devo mechanisms behind the appearance of seminal roots in maize

An important observation presented by Tai *et al.* (2017) sheds light on the genetic and regulatory mechanisms involved in the evolution of seminal roots in maize. The authors ran a transcriptome analysis (RNA-seq) on histological samples of wild-type and *rtcs*-mutant (deficient in seminal roots) embryos about a month after pollination (thus before seed dormancy). It is at this stage that seminal root primordia are initiated, right in the centre of the embryo, on the axis that connects the shoot apical and primary root meristems, in the scutellar node region (Box 1). More than 3000 differentially expressed genes were identified between wild-type and *rtcs* samples. Quite unexpectedly, these genes were significantly enriched with evolutionarily young genes. Therefore, given that seminal roots are a recent and specific acquisition of the teosinte/maize evolutionary lineage, Tai *et al.* (2017) suggest that there is a causal connection between the young genes and the presence of seminal roots.

But what exactly are *evolutionarily young genes*? In this context, these are non-syntenic genes, namely genes that lie away from their expected chromosome positions in the reconstructed gene order of the evolutionary ancestor. In the comparison between the two maize subgenomes and sorghum, non-syntenic genes amount to 40–50% of the maize transposon-filtered gene set (Schnable *et al.*, 2011; Schnable, 2015). The most likely explanation for their current position is that they were inserted recently (after the divergence of the teosinte/maize lineage from the sorghum one and after the maize-specific whole-genome duplication), by a copy-and-paste mechanism linked with transposon movement (Schnable, 2015). Tai *et al.* (2017) suggest that the genes currently acting downstream of the key root development transcription factor RTCS were recruited to the RTCS regulatory network from such non-syntenic young genes. At these genes, imperfect duplication events and other mutations probably caused sufficient reshuffling of regulatory elements (including RTCS binding sites) and/or coding properties that led to novel expression patterns and eventually to the initiation of seminal roots at the scutellum node. All this was probably soon reinforced by positive natural selection, given the adaptive value of seminal roots. The same authors proposed a similar hypothesis in a different paper in 2016 (Tai *et al.*, 2016), where they had observed that an Aux/IAA gene (*ZmIAA33*), a non-syntenic homologue of the seminal root gene *rum1*, was highly and specifically expressed in seminal roots.

In order to test this hypothesis, additional evidence could be gathered through genomic and morphological investigations in *Tripsacum*, which is the only other genus in the teosinte/maize (*Zea*) lineage. *Tripsacum* and *Zea* shared the same whole-genome duplication but subsequently had different histories of genomic rearrangement (Schnable, 2015). It would also be interesting to clone QTLs for number of seminal roots in maize (natural variation for this trait goes from none to more than ten: Hochholdinger, 2009). This should tell us if the variation within species (maize) and between species (maize vs sorghum) shares the same molecular basis.

## The relationship between non-syntenic genes and QTLs

The hypothesis proposed by Tai and co-authors is intriguing because most non-syntenic genes have been suggested to have little or no biological function (Schnable, 2015). Indeed, Schnable and Freeling (2011) observed that non-syntenic genes are nine times less likely to represent the causal loci behind mutations identified by classical genetics than syntenic genes. The only case in which allelic variation at non-syntenic genes has been shown to cause strong phenotypes is in relation to disease resistance. Disease resistance genes are overrepresented among genes found at non-syntenic locations among plants, as shown in a re-sequencing project of multiple rice genomes (Xu *et al*., 2011). Notably, the *Fusarium*-resistance gene recently cloned in wheat is non-syntenic (Rawat *et al.*, 2016).

These observations could also contribute to the interpretation of the molecular nature of QTLs. Most QTLs in a QTLome (the whole set of QTLs for a given trait in a given species; Salvi and Tuberosa, 2015) have subtle quantitative effects on phenotypes. The prevailing interpretation is that strong unfavourable QTL alleles are quickly purged by selection (natural or artificial), or quickly fixed if they have favourable effects (discussed in Salvi and Tuberosa, 2015). Thus, strong-effect QTLs are rare. When found, they have often been shown to correspond to genes already known based on classical mutations. This connection is also known as Robertson’s hypothesis, from its initial proposer (Robertson, 1985). The combination of the observation of Schnable and Freeling (2011) with Robertson’s hypothesis (1985) suggests that strong-effect QTLs should most frequently correspond to syntenic genes, whereas weaker-effect QTLs should correspond to non-syntenic ones, such as the genes identified in Tai *et al.* (2017). This is a testable hypothesis in the current era of high-throughput, high-resolution QTLome mapping, and the result could be useful in cloning genes of agronomic value.
